# Hā Ora: secondary care barriers and enablers to early diagnosis of lung cancer for Māori communities

**DOI:** 10.1186/s12885-021-07862-0

**Published:** 2021-02-04

**Authors:** Jacquie Kidd, Shemana Cassim, Anna Rolleston, Lynne Chepulis, Brendan Hokowhitu, Rawiri Keenan, Janice Wong, Melissa Firth, Karen Middleton, Denise Aitken, Ross Lawrenson

**Affiliations:** 1 School of Clinical Sciences, Faculty of Environmental and Health Sciences, Auckland University of Technology, Private Bag 92006, Auckland, 1142 New Zealand; 2 Waikato Medical Research Centre, Division of Arts, Law, Psychology and Social Sciences, The University of Waikato, Private Bag 3105, Hamilton, 3240 New Zealand; 3 The Centre for Health, PO Box 13068, Tauranga, 3141 New Zealand; 4 Te Pua Wananga ki te Ao Faculty of Māori and Indigenous Studies, The University of Waikato, Private Bag 3105, Hamilton, 3240 New Zealand; 5 Waikato District Health Board, Waikato Hospital, Private Bag 3200, Hamilton, 3240 New Zealand; 6 Lakes District Health Board, Rotorua Hospital, Private Bag 3023, Rotorua Mail Centre, Rotorua, 3046 New Zealand

**Keywords:** Lung Cancer, Māori, Barriers, Secondary care, Hospital, Diagnosis

## Abstract

**Background:**

Lung Cancer is the leading cause of cancer deaths in Aotearoa New Zealand. Māori communities in particular have higher incidence and mortality rates from Lung Cancer. Diagnosis of lung cancer at an early stage can allow for curative treatment. This project aimed to document the barriers to early diagnosis and treatment of lung cancer in secondary care for Māori communities.

**Methods:**

This project used a kaupapa Māori approach. Nine community hui (focus groups) and nine primary healthcare provider hui were carried out in five rural localities in the Midland region. Community hui included cancer patients, whānau (families), and other community members. Healthcare provider hui comprised staff members at the local primary healthcare centre, including General Practitioners and nurses. Hui data were thematically analysed.

**Results:**

Barriers and enablers to early diagnosis of lung cancer were categorised into two broad themes: Specialist services and treatment, and whānau journey. The barriers and enablers that participants experienced in specialist services and treatment related to access to care, engagement with specialists, communication with specialist services and cultural values and respect, whereas barriers and enablers relating to the whānau journey focused on agency and the impact on whānau.

**Conclusions:**

The study highlighted the need to improve communication within and across healthcare services, the importance of understanding the cultural needs of patients and whānau and a health system strategy that meets these needs. Findings also demonstrated the resilience of Māori and the active efforts of whānau as carers to foster health literacy in future generations.

**Supplementary Information:**

The online version contains supplementary material available at 10.1186/s12885-021-07862-0.

## Background

**Table:** 

*He tino honore mātou e whakanui ana mātou te kaupapa Hā Ora ki ngā iwi e whakatinana, e whakaora ai tēnei kaupapa ā Hā Ora ki a rātou kōrerorero. Kā whakawhetai mātou ki ngā whānau e kōrero ana te kōrero e hīkoi ana te hīkoi ki tēnei huarahi, kahuri kia rātou hoki e wehi atu ki rangi whetu ma ki tua o te ārai ano kia rātou e ora tonu ai me ngā uri e heke mai nei, nōreira he honore ano i a mātou ki te whakanui ēnei rangatira me ā rātou whānau ki a whiri whiri ā rātou kōrero kia tau te rangimarie, te aroha me te whakapono Paimarire.*	*We are very honoured to acknowledge those who shared their stories and brought to life Hā Ora. We are forever thankful and dedicate this to them and their families for embracing Hā Ora. For talking the talk and walking the walk. To those who have passed on, who reside among the many stars of the heavens, to those living who remain with us, and for the generations to come. Again, it is indeed a great honour for us to acknowledge these rangatira and their families. May peace, love and faith keep you safe. Paimarire.*

Lung cancer is one of the most common causes of death from cancer worldwide [1]. It is the leading cause of cancer deaths in Aotearoa New Zealand (NZ) with approximately 1650 deaths per year [2]. In particular, Māori (Indigenous peoples of NZ) account for 16.5% of the NZ population [3], and have both higher incidence and poorer survival rates for lung cancer compared to non-Māori [4–8]. For instance, mortality rates for Māori from lung cancer are 2.6 times greater than in NZ Europeans [2]. The persisting health disparities between Māori and non-Māori in NZ are of particular concern [9, 10].

Diagnosis of lung cancer at an early stage can allow for curative treatment [11, 12]; however, lung cancer is typically diagnosed at a late stage when treatment tends to be palliative [13]. Barriers to early diagnosis can occur at various stages throughout the diagnostic pathway. Barriers in primary care for Māori in particular are primarily related to General Practitioner (GP) – patient relationships, the health literacy of patients and health providers, and factors such as cost, symptom presentation and delayed diagnosis [14, 15]. Consequently, previous NZ research indicates that many lung cancer patients initially present to secondary care through the emergency department (ED) rather than by referral from GP to a respiratory specialist [16]. However, this pathway also presents barriers to patients. Walton and colleagues [17] indicate that barriers to early diagnosis for patients (both Māori and non-Māori) presenting directly to a hospital ED involved disparities in access to services, and disparities and delays relating to processes of care. Māori lung cancer patients in particular, are more likely to be admitted via ED and tend to have different treatment plans to non-Māori [13, 18]. However, further research is needed to identify the barriers to early diagnosis of lung cancer in secondary care for Māori.

This article discusses the findings from a broader project entitled Hā Ora: Improving early access to lung cancer diagnosis for Māori and rural communities. The objective was to explore the barriers to early presentation and diagnosis of lung cancer, as identified by Māori patients, whānau (families) and primary healthcare providers in the Midland region of NZ. This paper reports on additional findings from the Hā Ora project that relate to the barriers and enablers to early diagnosis and treatment of lung cancer that are specific to the secondary care setting.

## Methods

This qualitative research used a kaupapa Māori methodological approach. Kaupapa Māori approaches emphasise local cultural contexts and self-determination by prioritizing Māori history, development and aspirations [19]. As such, kaupapa Māori initiatives have been associated with improved health outcomes and engagement for Māori (e.g. [19–22]). This approach enabled us to interrogate systems of power and dominance within the health system, and to illustrate the agency and resilience of the Māori communities who collaborated on the research.

The team carried out hui (focus groups/meetings) with community members and healthcare providers in five rural localities in the Midland region. All community hui (CH) were organised in conjunction with key Māori stakeholders in each community and followed local tikanga (protocols). Participants were recruited using ‘snowball’ sampling. Personal contact with local stakeholders and/or primary care providers was followed by the distribution of written materials to each community, inviting them to participate in hui. Kidd and colleagues’ [23] publication provides a detailed discussion of the methods used in this research. CH were led by Māori members of the Hā Ora team (JK and AR) and occurred at local meeting rooms or marae (Māori meeting houses), whereas primary healthcare provider hui (HCP) was facilitated by RL, and took place at local GP practices. All facilitators had extensive experience in community engagement.

Hui data was recorded as field notes and via an audio recorder. Audio recordings were transcribed and anonymized. Transcripts and field notes were thematically analysed [24]. Coding was carried out on qualitative data by two researchers (JK and SC) independently and then together, to ensure a rigorous analysis process. Accordingly, codes were developed into categories, and the categories were allocated into two broad themes, each comprising several sub-themes. Rigour was also considered based on the COREQ guidelines ( [25]; also see completed COREQ checklist available as supplementary material).

## Results

A total of nine CH and nine primary HCP hui were carried out by the Hā Ora team. Each CH comprised of between 8 and 21 participants, which included cancer patients, whānau, and anyone else in the community who may be affected by (lung) cancer. Each HCP hui comprised 1–6 staff members at the local primary healthcare centre or General Practice, which included the GP, nurses and/or other staff. Overall, the CH comprised 108 participants, and the HCP hui comprised 27 participants in total.

**Fig. 1:**
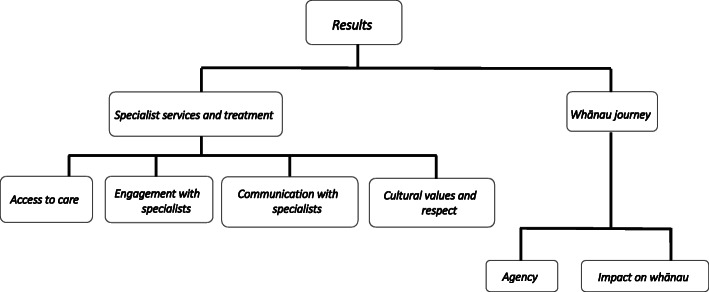
Diagram of themes and sub-themes comprising the results of the present study

Data analysis generated two key themes relating to secondary care settings: “specialist services and treatment”, and “whānau journey”, which will be discussed in the following sections of this article. Figure 1 shows how the results in terms of the themes and sub-themes are structured.

### Specialist services and treatment

The barriers and enablers that participants experienced in relation to specialist services and treatment are classified by four sub-themes: access to care, engagement with specialists, communication with specialist services and cultural values and respect. Here, we present both community and HCP accounts together, to demonstrate that the issues raised are experienced by both whānau and HCPs.

#### Access to care

Many participants highlighted barriers related to secondary care that they experienced leading up to, or immediately following initial diagnosis. These barriers included long waiting times and GPs having to advocate for patients:

*We have to travel across to* [the hospital that is approx. one hour drive away]*. Then sitting there waiting for four hours then* [our whānau] *get seen…. It’s almost, may as well be a whole day, especially with young kids*. (CH#1)

*My whānau, they’ve had to wait almost an hour for an ambulance to get out to* [my uncle’s place]*. And we lost that uncle*... (CH#1)

*I had a patient who came in with back pain, referred for a chest x-ray, it was three weeks till the patient could be seen for a CT. Even that was after I had a conversation with the respiratory physician. GPs need to navigate it. You can’t just send a referral through and just leave it.* (HCP#6)

*Referral depends on symptoms, background of age, smoking etc. I’ve sent through lots of requests for high suspicion of lung cancer. The majority are acted upon quickly, some were declined. As the person dealing with the client, I feel like the request should go ahead and not be declined. If it’s haemoptysis, I tend to do a chest x-ray, and then follow up to see if it’s cleared. At the back of your mind you wish they’d approve the CT referral. They have a category for ‘high suspicion’* [of cancer]. *So the referral being approved depends on the hospital’s interpretation of the background information we provide.* (HCP#5)

All the primary care providers we interviewed also described difficulties with secondary care, particularly related to accessing CTs for their patients. While some described frustration with the system, others employed specific tactics to get past the barriers to having a CT referral accepted:

*We use* [respiratory e-referral pathway]. *You have to meet criteria to get a CT. For example, a chest x-ray abnormality, haemoptysis, pleural fusion, or something like that. If you don’t have any of these, then you can’t refer for a CT. So I just lie. The system has improved from before, but it’s still a hoop* [we have to jump through]*. Now I can just ring the radiologist that I know and get an endorsement, which lets me refer for a CT. Still, I’ll do a chest x-ray first but if we can’t find any of the above criteria, I’ll just lie and tick the box.* (HCP#2)

#### Engagement with specialists

Patients and whānau also discussed negative and stressful experiences relating to their interaction with the specialist. This participant’s story relates to waiting to hear what his wife’s diagnosis would be, during a manifestly unsatisfactory specialist appointment:

*We sat there absolutely petrified, waiting to squeeze every little bit of information they had in that little half an hour session. A secretary from upstairs came down twice to present some other patient’s case. And it just broke… I was just angry after that. …I thought we were going to get their devoted attention.* (CH#6)

The quote below is representative of several participants’ frustration about how information was shared with them and the differences between what clinicians and whānau understand by specific terms such as radiotherapy and chemotherapy:

*For me it’s about sensitivity. Or the lack of it. My brother, when he was diagnosed, they said, why don’t you go through 6 week radiotherapy. And after that he came home, we get a phone call, and the phone call says well if it doesn’t work out you can go on chemo. From the best case of beating it, to the next step might be chemo? And to Māori chemo means, you’re just about to get pushed off a cliff. It’s a death sentence. They just told him you’re at the cliff edge. And he just said, no. I might as well carry on drinking, smoking and dying that way… From saying you’ve got cancer, it can be beaten! And then they say oh you’re going on chemo. It’s ridiculous.* (CH#6)

Specifically, patients and whānau indicated that HCPs needed to give them a little hope, even if they had exhausted their treatment options and were instead looking for symptom relief. In the quote below, the participant describes finding a remedy (ginger) for ongoing nausea on Google:

*My husband had 3 rounds of chemo and it didn’t work, and then they said “sorry”. That was pretty blunt. “Sorry, can’t do anything else”. What really annoyed me was after being with them for that long, they didn’t have anything else. They didn’t even - or couldn’t or wouldn’t - refer you to anything natural. To give it a go. Cause what have we got to lose? Where does he go from here? Surely you can send us somewhere. Give us some hope. It was old Google that helped us in the end. It didn’t help us fully. But we got on there and had a look at what was being offered naturally.* (CH#6)

The context of the previous two quotes highlights the ineffective interactions that frustrated patients and whānau. In the first quote the specialists did not identify the whānau gap in knowledge, so the whānau believed that radiotherapy was an almost certain cure and chemotherapy was a death sentence. The result of this misunderstanding was that the patient did not continue with treatment. In the second, the specialist appeared to be focusing on the limitations of a narrow area of treatment and did not understand that whānau expected to be guided more generally about what they could do for the patient after curative treatment was unsuccessful. It is interesting also that the whānau did not appear to receive any information about accessing palliative care services.

#### Communication within specialist services

A lack of communication among specialist services within a hospital as well as across hospitals or District Health Boards (DHBs) was another key concern that whānau discussed:

*The left arm didn’t know what the right arm was doing, so the communication in the same ward didn’t happen. An example, was that my husband had a drug rash. A real bad drug rash from a particular type of antibiotic. So bad that he couldn’t even lie on the bed. Two days later, a nurse turned up with exactly the same drug, and she put it up there and I said, “oh no no no, you’re not giving him that. He’s allergic to that”. She took the chart and said, “not on here”. I said, “I don’t care. You’re not giving that to him”. Another doctor said, “no water for this patient”. And then when the specialist had his days off, he* [husband] *gained something like 8 kilos in about 2 days. Too much water. So again, I intervened and said, “no more. Stop”. It’s not rocket science.* (CH#6)

*I didn’t know that* [hospital A] *couldn’t share information with* [hospital B]. *So like that’s really frustrating… It’s like they don’t talk to each other…. And the cancer centre there, where he did his PICC line a couple of times. The processes are different. Why are they different? Why isn’t there a standard for something like flushing those PICC lines?* (CH#6)

#### Cultural values and respect

Participants in the CH discussed the importance of having hospitals value tikanga (customs and values) processes, where their experiences highlighted gaps in the health system’s ability to cope with tikanga Māori. Key points discussed were an awareness of the involvement of wider whānau in the specialist care journey (rather than a focus on only the patient) and showing respect to elderly patients:

*. . . the whole tikanga within the process. Knowing that we come with many whānau members, children, aunties, uncles, everybody wants to come, so shared rooms don't really meet our needs. Having somewhere for our children, so that they’re not being a distraction or a hōhā* [nuisance]*, but that they need to be there and their koro’s* [grandfathers]*, their nans, they need them there. … This is part of your healing process, this is what is going to make it better for you. ‘Cause in here it’s a positive outlook for them and that will improve their treatment response.* (CH#1)

Some whānau described experiences of racism that resulted in fierce protectiveness of the patient. Most stories stemmed from institutional rules and expectations about how patients and whānau should behave that were not conducive to a Māori form of manaaki (respect/care):

*Our koros and our kuias* [elders]*; their mana* [status/authority] *gets tramped on. Their wishes don’t get respected. If you are tūturu to your Māori-ness* [everything is subsumed by your Māori identity]*, you know that the whānau looks after their own. And when they are sick and they go to the hospital, that all goes out the window. It becomes, excuse me, the white man’s rule. There is no negotiating. You do it this way or you get out. I don’t get out. I got a mouth. And our old people, they don’t want other people wiping their bums, washing them. That is what keeps their mana intact, having that respect. . . . Their* [HCP] *job is to look after the tinana* [body]*, but you need to look after the wairua* [spirit/soul] *too. Because that’s what keeps the person going.* (CH#2)

A participant also described how her mum’s response of quiet listening and processing when receiving her diagnosis, was misinterpreted as her being deaf:

*I walked into the room and the doctor was yelling, speaking incredibly loud. I said “you know what? She’s not deaf!”, “oh oh! I’m sorry!”, the assumption that she was deaf, but she actually had a scarf around her head, and I think it was because she didn’t respond to him. She’d just been told she had terminal cancer, and I think she really wanted to just bawl! But she just sat there looking, in her seat … the assumptions that people have … if they’re old, if they’re Māori, if they’re female, there’s this whole…yeah. And without them realizing, this bias against what’s presented in front of them. We have to deal with that. The system has to deal with that. I think that’s something the organization has to deal with. That institutional racism, unconscious bias and the attitudes that derive from that.* (CH#7)

### Whānau journey

Barriers and enablers in secondary care relating to the whānau journey into and through cancer comprised two subthemes: agency and the impact on whānau.

#### Agency

Participant accounts demonstrated various instanced where whānau enacted agency when caring for patients by taking the initiative at various points in the cancer care journey, which served as enablers for patients. Here, agency is enacted when whānau advocate for and act on behalf of patients. For instance, as carers, whānau were proactive, and many recounted how they had to actively fight the system and advocate for patients in secondary care:

*My brother, he actually took me on. Because I was too ill to email and fight for my rights so he took my email and started to say look when am I gonna get treatment? And he just happened to be rung to say we’re having this* [PET] *scan… And I was in there and he rung the oncologist and said my sister is in there now having a PET scan, please if you have a spare bed can she go in. And I went straight from there up, through my 1st round of chemo. But you have to fight as well for your treatment. And when you’re too ill, get someone who can talk to the pathologist or radiologist, to say when is it going to happen.* (CH#5)

Whānau ensured that even their children were part of every step of the patient’s journey so that they learned how to manage and navigate the healthcare space in the future:

*I will tell my kids exactly what’s happening so they are aware and they understand . . . even though they were only young, to me they needed to know. So they could see all the stages that their grandparents were going through. They seen the hair loss, they seen the sick, they seen the weak, the frail, they seen all of that. And they’ve sat in the chemo treatments talking to them. Getting food for her, helping in whichever way they could. They came to all the appointments and everything. So they knew exactly what was going on. And that’s been a massive journey. I still think my kids are richer for that, having spent time with their whānau and their grandparents, and richer for being involved in those processes so that if ever they come across friends and family* [who get sick]*, they understand and they know. So they can tautoko* [support] *and help.* (CH#2)

#### Impact on whānau

As discussed, whānau are central enablers in the diagnosis and treatment journeys of Māori lung cancer patients. Accordingly, impacts on whānau members, who often took on the carer role, are significant. Such impacts stretched beyond healthcare, into other areas such as living situations and work:

*I gave my job up to look after him. Tried to find a job that would do me from home. And I did, it’s doable, and if you stick together you’re alright. As long as you have one strong person in your family you'll be right. You just chug on.* (CH#1)

*So we made that decision to come home* [from abroad] *. . . We managed to get a rental . . . and I went through all the processes, doc’s visits, chemo treatments that sort of stuff with my mother-in-law…* (CH#2)

## Discussion

Many of the findings of this study were consistent with previous research identifying secondary care barriers to the diagnosis of lung cancer that led to delays at the primary-secondary care interface [17]. Such barriers include delays for referrals (to diagnostics and/or specialist assessments), declined referrals from primary care (despite being flagged as a ‘high suspicion case’) and long waiting times in accessing specialist care. These delays were not specific to the lung cancer pathway, but also other forms of cancer [17, 26]. Findings are symptomatic of a health system which is under stress including having a shortage of senior medical staff [27]. For a number of years GP referrals to specialist care have had to be rationed with some patients not being able to be seen. Similarly, GP access to diagnostic tests such as CT are poor in NZ compared to other countries [28]. Our study indicates that barriers in secondary care were experienced by patients as well as GPs. In addition to long waiting times, a lack of communication between departments and/or hospitals was identified by some participants. Here, GPs highlight the need for coordination and collaboration between HCPs when working with patients and whānau. Such delays and hurdles caused barriers to earlier diagnosis and treatment of lung cancer. GPs admitted to finding workarounds such as sending all referrals through as high suspicion of lung cancer, drawing on endorsements from specialists, or falsely applying the criteria in order to ensure prompt acceptance of their referral.

This study draws attention to the importance of tikanga and the complex involvement of whānau through a Māori patient’s lung cancer journey. An acknowledgement of, and adherence to tikanga is certainly vital to ensuring patient engagement and treatment uptake, but also to showing basic respect for Māori patients and whānau in secondary care. The importance of tikanga in the broader healthcare system is not a new finding. For instance, research not specific to the field of lung cancer highlights the importance of whakawhanaungatanga, or culturally meaningful connections and respect in secondary care [29–31]. For other groups, similar cultural understanding and respect is required. This can be a challenge for a health system which is under stress and relies heavily on the use of clinical staff, especially doctors from other countries. Yet, it is an issue that needs to be addressed. While one solution to this can be the provision of intensive cultural training for clinical staff, another solution that has been used is to employ cultural navigators who provide a bridge between patients and staff [32–34]. The Bay of Plenty DHB in New Zealand, for instance, have recently engaged navigators who support Māori whānau through their secondary care journey [35]. Additionally, a workforce strategy has been proposed to train more New Zealanders, especially Māori and those from a Pacific Island (PI) background, in medicine and other clinical roles to better meet the needs of an increasing Māori and PI population [36]. It should be recognised that this strategy will take many years before the current proportion of 3.5% of Māori medical practitioners begins to mirror the proportion of Māori in the population.

Overall, Māori patients are entitled to receive culturally appropriate and respectful care and interactions from secondary care providers and staff. This is important, particularly given the fact that Te Tiriti o Waitangi (the Treaty of Waitangi) has been poorly upheld in NZ, particularly in health, resulting in the unequal distribution of the determinants of health and inaction for Māori in the face of need [37]. As such, in the present context, engaging in kaupapa Māori processes such as whakawhanaungatanga (making meaningful connections), conversing in te reo (Māori language) and taking time to fully answer questions is an avenue to redress this issue. Such steps have also been discussed as modes of increasing provider-patient engagement and to providing higher quality interactions in secondary healthcare [31].

An acknowledgement of whānau involvement in a Māori patient’s lung cancer journey comes hand-in-hand with adherence to tikanga and culturally safe care [38]. Research by Jansen and colleagues [30] for instance, demonstrates the vital role that whānau play to support and advocate for patients through their hospital journey. Our study sheds further light on the sheer complexity of whānau involvement, which goes beyond the immediate hospital setting, and the broader implications of being a carer, that are incorporated into their everyday lives. Our participant accounts demonstrate the resilience and agency of patients and whānau to learn, support and advocate for each other. Such demonstrations of agency and health literacy call into question the mainstream misinterpretation of (Māori) patients as being helpless, lacking initiative, not being compliant, and disinterested [39, 40]. This research contributes to the mounting kaupapa Māori evidence in other areas of healthcare (e.g. [31, 41–45]) demonstrating that there needs to be an acknowledgement of the proactive initiatives that Māori engage in to care for their whānau. Participants of this study were also actively teaching or exposing their tamariki (children) to the processes involved in caring for the unwell, thus fostering health literacy in the next generations. These findings demonstrate that a lung cancer journey does not simply involve singular units of individual patients. Rather, a lung cancer journey is taken by entire whānau networks, who go to extreme lengths to support and care for each other, today and also in the future.

The findings of this study raises a number of issues. The NZ healthcare system overall, but certainly in secondary care, needs to ensure that the needs of Māori patients and whānau are met. Primarily, health systems must work towards consistently increasing Māori patient engagement, by supporting patients and providing higher quality, culturally safe care to patients as well as whānau. Additionally, drawing on the Health and Disability Commission’s code of rights [46] and research into culturally safe healthcare [37, 47, 48], the health system should look at ways of supporting staff, through more cultural training and the provision of cultural navigators that support patients and staff. In the longer term there needs to be a review of the health workforce strategy that reduces the shortages in the system and helps provide a workforce that more closely mirrors the communities it serves. There also needs to be an improvement in communication between HCPs, and a national standard of care (e.g. in relation to processes for administering PICC lines) irrespective of DHB or region. These issues have been recognised and will be something for the newly formed Cancer Control Agency – Te Aho o Te Kahu to address [49]. Finally, providing GPs with the ability to request for diagnostic tests such as CT scans could also be an avenue to speed up the diagnostic pathway, and overcome barriers in early diagnosis of lung cancer in secondary care.

This study had several strengths and limitations. The strengths of this study include an inherently kaupapa Māori approach, and a holistic view of barriers within secondary care, as perceived by patients and whānau, as well as primary healthcare providers. A limitation of this study was that it has not included the perspective of the secondary care services in the research. Future research could explore the wider perspectives of secondary care services relating to barriers and enablers to diagnosis and treatment of lung cancer, and may provide further information that was not captured in this study.

## Conclusion

This research provided insight into barriers as well as enablers experienced by Māori lung cancer patients and whānau in secondary care. While some barriers and enablers may be more applicable to the NZ context, findings can be relatable and thus applicable to other Indigenous and/or minority groups globally. Lessons learned include the need to improve communication within and across healthcare services; an understanding of the cultural needs of patients and whānau and a health system strategy that meets these needs. We should also acknowledge the resilience and agency of Māori and the active efforts demonstrated by whānau to foster health literacy in the future generations. Other lessons include better access for diagnostic services for primary care practitioners and greater standardisation of care between different secondary care providers.

## Supplementary Information

**Additional file 1.**

**Additional file 2.**

**Additional file 3.**

## Data Availability

All data generated or analysed during this study are included in this published article.
