# Supplementary material for: Hā Ora: secondary care barriers and enablers to early diagnosis of lung cancer for Māori communities
Source: BMC Cancer. 2021 Feb 4;21:121. doi: 10.1186/s12885-021-07862-0 (PMC7863263; doi:10.1186/s12885-021-07862-0)
Supplement: Supplementary file 1 — Additional file 1. [file 12885_2021_7862_MOESM1_ESM.docx]

| **No** | **Item** | **Response** |
| --- | --- | --- |
| *Domain 1: Research team and reflexivity* | | |
| 1 | Interviewer/facilitator | Methods, paragraph 2 |
| 2 | Credentials | All have PhDs. However, this is not applicable to the methods section of this manuscript |
| 3 | Occupation | All are academics/researchers |
| 4 | Gender | A1 and A3 are female and A11 is a male. However, this is not applicable to the methods section of this manuscript. |
| 5 | Experience and training | Methods, paragraph 2 |
| 6 | Relationship established | Methods, paragraph 2 |
| 7 | Participant knowledge of the interviewer | n/a |
| 8 | Interviewer characteristics | n/a |
| *Domain 2: Study design* | | |
| 9 | Methodological orientation and Theory | Methods, paragraph 1 |
| 10 | Sampling | methods, paragraph 2 |
| 11 | Method of approach | methods, paragraph 2 |
| 12 | Sample size | Methods, paragraph 3 |
| 13 | Non-participation | n/a |
| 14 | Setting of data collection | Methods, paragraph 2 |
| 15 | Presence of non-participants | n/a |
| 16 | Description of sample | methods, paragraphs 2 and 3 |
| 17 | Interview guide | Broad themes and prompts were provided by the facilitators. However, for the most part, the participants directed the focus group conversations. |
| 18 | Repeat interviews | no |
| 19 | Audio/visual recording | methods, paragraph 4 |
| 20 | Field notes | methods, paragraph 4 |
| 21 | duration | Focus groups were 1-2 hours in duration |
| 22 | Data saturation | n/a |
| 23 | Transcripts returned | Broad themes were re-checked for accuracy with participants at the focus groups. |
| *Domain 3: Analysis and findings* | | |
| 24 | Number of data coders | Methods, paragraph 4 |
| 25 | Description of coding tree | n/a |
| 26 | Derivation of themes | Themes were derived from the data. Methods, paragraph 4 |
| 27 | software | n/a |
| 28 | Participant checking | yes |
| *Reporting* | | |
| 29 | Quotation presented | Yes (see results section) |
| 30 | Data and findings consistent | yes |
| 31 | Clarity of major themes | Yes (see subheadings in results section, and statements relating to ‘most’ or ‘all participants’) |
| 32 | Clarity of minor themes | Yes (see accounts relating to ‘some participants’ in results section) |
