# Supplementary material for: Hā Ora: secondary care barriers and enablers to early diagnosis of lung cancer for Māori communities
Source: BMC Cancer. 2021 Feb 4;21:121. doi: 10.1186/s12885-021-07862-0 (PMC7863263; doi:10.1186/s12885-021-07862-0)
Supplement: Supplementary file 2 — Additional file 2. [file 12885_2021_7862_MOESM2_ESM.docx]

**SS2 Interview Guide: Hui/focus groups**

Two focus groups will be conducted in each of the intervention localities (n=12) with Māori who self-identify as being at risk of lung cancer and their whānau.

**You do not have to ask all the questions. Let the group lead the conversation. However, if the conversation stalls or goes off on a tangent, you could use these questions as prompts.*

(1st meeting) Introduction - **Whanaungatanga**

- Karakia
- Thank participants for their time and agreeing to participate
- Introduce self/background – the same for all participants
- Explain aims of research and interview
- Explain use of the whiteboard
- Verbally go through participant information sheet, and answer any questions/clarify any doubts
- Give participants information sheets and ask for verbal consent
- Have sign in/address sheets doing the rounds so people can indicate their presence and request follow-up information

Could we start off by talking about what you all know about lung cancer?

Prompts:

- Causes
- Symptoms
- Risks
- Prognosis – is there any hope?
- Treatments

**Who or where would you go to first, if you’re worried about your risks or symptoms?**

**How do you decide if it’s time to see a doctor?**

Prompts:

- Risk factors
- Whanau/community member suggestion
- Critical events or being really sick
- Health literacy
- Public health campaigns

**What is it like to see a doctor?**

Prompts:

- Geographical accessibility
- Making the appointment
- Feeling comfortable/uncomfortable, embarrassed/shy/whakama, scared/racism
- Paying for it
- Taking time off work
- Whānau support or lack thereof

What kind of things help you decide to see a doctor, or put you off from going?

Prompts:

- Barriers
- Enablers
- Have these happened, or is it what the participants expect to have happen?
- Whakama – e.g. do they delay or avoid going if they’re smokers or overweight
- Supportive care or lack thereof

**What advice would you give to someone else starting out on this path?**

**Is there anything you would like to change about the way people at high risk for lung cancer are cared for by health services?**

***Prioritise main points of information on the whiteboard and encourage further input from the participants***

- Is there anything you would like to bring up or think should have been discussed?
- Do you have any further questions about this study?

***Reminder***

- Address/contact details to send through summary report of research - *if required*
- Karakia whakamutunga
