# Supplementary material for: Hā Ora: secondary care barriers and enablers to early diagnosis of lung cancer for Māori communities
Source: BMC Cancer. 2021 Feb 4;21:121. doi: 10.1186/s12885-021-07862-0 (PMC7863263; doi:10.1186/s12885-021-07862-0)
Supplement: Supplementary file 3 — Additional file 3. [file 12885_2021_7862_MOESM3_ESM.docx]

**SS3 Interview Guide: Interviews and/or focus groups**

Interviews and/or focus groups (depending on the preference of the participants) with up to 20 individual primary care practitioners in each location (consisting of Māori providers, GPs, primary care nurses and where appropriate rural hospital staff).

**You do not have to ask all the questions. Let the group lead the conversation. However, if the conversation stalls or goes off on a tangent, you could use these questions as prompts.*

(1st meeting) Introduction - **Whanaungatanga**

- Thank participants for their time and agreeing to participate
- Introduce self/background (the same for all participants – in hui, if applicable)
- Explain aims of research and interview
- Verbally go through participant information sheet, and answer any questions/clarify any doubts
- Give participants information sheets and consent forms (or verbal consent based on situation)
- Consent for turning Dictaphone on

What is your experience of working with a suspected diagnosis of lung cancer?

Prompts:

- Frequency
- Confidence in own processes

What challenges do you have when you’re treating someone you think may have lung cancer?

Prompts:

- Ability to refer effectively
- Access to diagnostic and specialist services
- DNAs and ability to convince patients to access care
- Being kept in the loop after referral
- Patient barriers (e.g. cultural issues, mistrust of medical system/GPs)

What challenges do you think your patients face when they’re at risk of lung cancer?

Prompts:

- Geography
- Cost
- Cultural acceptability of services
- Time off work
- Continuity of care – existing relationships or lack of

**What would help you to provide better care?**

Prompts:

- Information
- Resources
- Time

***Summarise main points of interview and encourage further input from the participant***

- Would that be an accurate summary?
- Is there anything you would like to bring up or think should have been discussed?
- Do you have any further questions about this study?

***Reminder***

- Address/contact details to send through summary report of research - *if required*

__________________________________________________________________________________________________________________________________________________________________________________________________________________________________________________________________________________________________________________________________________________________________________________________________________________________

***Thank you so much for your time***
